# CT103A, a forward step in multiple myeloma immunotherapies

**DOI:** 10.1097/BS9.0000000000000068

**Published:** 2021-04-27

**Authors:** Jie Xu, J. Joseph Melenhorst

**Affiliations:** a Center for Cellular Immunotherapies, Perelman School of Medicine at the University of Pennsylvania, Philadelphia, PA 19104, USA; b Department of Hematology, Shanghai Institute of Hematology, Ruijin Hospital affiliated to Shanghai Jiao Tong University School of Medicine, Shanghai, PR China

Even with the most potent, modern therapies, multiple myeloma (MM) remains a largely incurable disease. The reprogramming of a patient's T-lymphocytes with a cancer-associated antigen-specific chimeric antigen receptor (CAR) has improved the outcome of patients with this debilitating disease. B cell maturation antigen (BCMA), a marker almost exclusively expressing by plasma cells,^1^ is the most attractive target for designing CAR-T cells against MM. It is also largely hoped to be the next FDA-approved CAR-modified cellular product after CD19 CAR-T cells’ achievement. The most promising anti-BCMA CAR T cell products, developed by Bluebird Bio and Nanjing Legend, have been evaluated in large multi-site trials.^2,3^ However, whether durable remissions will be achieved with either product remains to be answered, but the non-human origins of the BCMA-targeting antibody fragment(s) may present an obstacle in achieving.^4^

In the latest issue of Blood, a group from China reported results from a Phase I clinical study which utilized a fully human anti-BCMA CAR (CT103A) to treat 18 relapse/refractory MM patients.^5^ Compared with other BCMA CARs reports where overall response rate (ORR) was 78% to 89% with a 32% to 53% of complete remission (CR),^6^ CT103A achieved an ORR of 100%, with 72.2% of patients reaching CR or stringent CR (sCR), and 88.9% achieved very good partial response (VGPR) or better. In the first month after CAR-T cell infusion, all evaluated cases’ bone marrow (BM) were detected negative for minimal residual disease (MRD) by flow cytometry. No anti-drug antibody (ADA) was detected, except in one case with an unclear reason. With a 394-day median follow-up, the median duration of response reached 325 days for all subjects. Both median progression-free survival (PFS) and overall survival (OS) have not reached 1 year. This therapeutic effectiveness is highly impressive.

Several phenomena are shared in MM CAR-T cells treatment. These common observations trigger us to think deeply since they provide accumulative evidence for further improvement in clinical scheme design and laboratory manufacturing.

First, the response to CT103A proceeds over time. Most of the patients entered into the minimal response (MR)/partial response (PR) stages early after infusion, and achieved VGPR or better later on. As the authors described, the ORR was 77.8% in the first 2 weeks, raising up to be 88.9% at 1 month and achieving 100% overtime at the best response. This phenomenon is commonly observed in MM^7,8^ and may be explained as follows: 1) A delayed depletion of serum monoclonal immunoglobulin and/or light chain, key parameters for disease evaluation, often occurs after BM remission; 2) localized myeloma lesions are surrounded by cellular and non-cellular compartments which render it slow for CAR-T cells trafficking and killing. Thus, cellular analysis based on BM puncture or tissue biopsy is more accurate to reflect MM status and assess CAR-T cells effectiveness.

Second, the extramedullary disease remains an unfavorable factor for anti-BCMA CAR T cell treatment. Four out of 18 patients on the current study eventually relapsed (1 of 4) or progressed with disease (3 of 4). Notably, extramedullary myeloma (EMM) was correlated with a shorter PFS which was only 20% at 1 year. Flow cytometry-based examination demonstrated a remarkable reduction of BCMA expression on two evaluable relapsed BM samples. A multitude of factors has been uncovered over the year that explains resistance to CAR T cell therapies, including poor immune function of apheresed T cells, low affinity of CAR, low or loss of targeted antigen, hostile tumor microenvironment (TME), and host immune rejection.^9^ In this study, reduced target antigen expression levels combined with impaired accessibility and expansion of CAR-T cells within TME might be the two key causes for a high-frequency relapse/PD in EMM. It offers an indication that CAR-T therapy, for EMM patients, is more likely to serve as a bridge that should be connected to timely maintenance therapy or allergenic transplant.

Third, as shown in other trials, there appears to be no correlation between the infusion dose-response to the therapy, underscoring previous findings that the memory component of the therapy is the key driver of clinical efficacy.^10^

Fourth, the use of steroid for CRS management in 66.7% patients has no impact on response. This observation is in line with a previous study which demonstrated that <10-day usage of steroid had no impact on the CAR T cell efficacy.^11^ In addition, this study described the AEs occurring 2 months later. Infectious complications were a major AE on this and other BCMA-targeting CAR T cell trials. This long-term impact of post-infusion plasma cell depletion is a major problem that requires periodically (monthly is recommended) intravenous immunoglobulin supplementation until normal plasma cell production has recovered. For better understanding, the deficiency of antibody-producing plasma cells post anti-BCMA CAR-T cell therapy, prospective or retrospective studies about the duration time of plasma cell deficiency in patients with favorable outcome, the relevant follow-up diseases, and the corresponding supportive-care approaches based on a large number of cases are highly expected.

Interestingly, 4/18 CT103A-treated had not responded to prior murine anti-BCMA CAR T cell therapy. Remarkably, all four patients responded to the fully human BCMA-specific CT103A therapy, with three on-going CRs and one VGPR. More interestingly, both murine and human CAR transgenes were monitored in this study. Around day 10 after infusion, with a high peak of human CAR transgene expansion, murine CAR in three patients resurged albeit at 2-3 log lower levels, followed by the rapid disappearance of murine CARs in 1/3, the maintenance of the murine CAR in the second patient, and the gradual loss in the third. In all three subjects, CT103A was maintained at high levels. Obviously, tumor depletion is accomplished by human CAR-T cell expansion, but it leaves us two indications: 1) whether the murine CAR is expressed at all in this gene marked population, and if expressed, whether the murine origin of the scFv resulted in the rapid immune elimination of this population and 2) whether murine CAR re-expansion is due to those same cells potentially expressing the human CAR, or the possibility that those cells re-expanded in response to TCR triggering. Further studies are needed to elucidate these.

Given that the median follow-up time is approximately 1 year, long-time observations on prognosis and lab tests are greatly required.

In the clinical setting, other than side effects, long-lasting response to CAR T cell therapies remains a challenge. We notice that a survival plateau commonly exists in anti-CD19 CAR-T cells for B-cell malignancies treatment.^12–14^ Usually, PFS plateau occurs at 12 to 20 months after infusion, which indicates a persistent good response and even a probably cure. In this study, 61% patients have on-going deep response at the cutoff point. How durable can those CR patients be? It is worth keeping follow-up for long enough. Trials in a larger number of subjects are highly looked forward for safety and efficacy validation. It is also worth further evaluating on a cohort with less limited enrollment criteria.

With respect to bedside-to-bench data, CAR transgene detection remains of interest to look into CT103A persistence. The long-lasting surviving CAR-T cells deserve to be analyzed by the means of high-throughput genomic/transcriptomic sequencing, multi-parameter immunophenotyping, and metabolomic screening to identify biomarkers and mechanisms of response and resistance. Furthermore, this study examined soluble BCMA levels as done by others on similar trials,^15^ which may serve as a more specific and sensitive biomarker of myeloma response to CT103A. Long-term monitoring of sBCMA should be helpful to get a quick sign of disease progression, particularly more valuable in non-secretary MM.

In conclusion, the pilot trial of CT103A marks a forward footprint in the field of MM cellular immunotherapy. Humanized CAR engineered product has brought improvements but also leaves a series of indications that motivate us to optimize the utilization of anti-BCMA CAR-T cells. The ice of hurdles in MM CAR-T cells treatment is eagerly awaits to be melted by more strategic fireworks (Figure 1).

**Figure 1:**
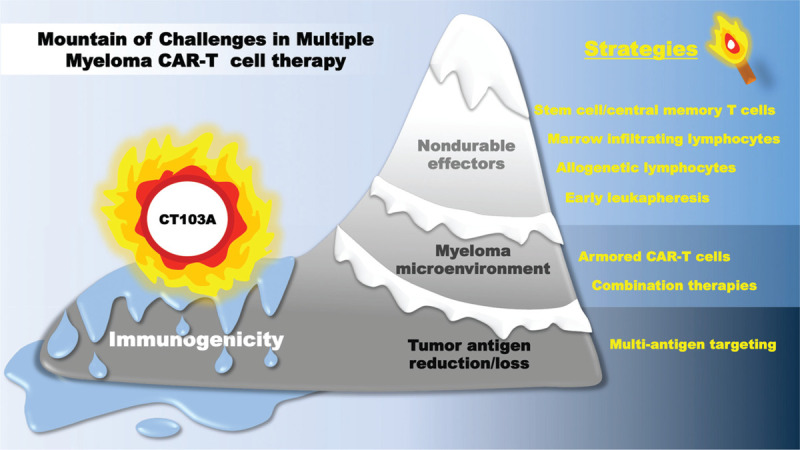
Challenges and solutions of CAR-T cell therapy in multiple myeloma. Host immune responses against non-human CAR sequences are considered to be a resistance mechanism for CAR-T cells to achieve durable remissions in myeloma. CT103A is a fully human anti-BCMA CAR engineered T cell which overcomes this big hurdle and thus induces and potentially sustains complete remissions. However, there still remain other challenges of CAR-T cells in MM, including T cell- and tumor-related challenges, including the underlying health status of the immune compartment and the immune-suppressive tumor microenvironment in myeloma. Novel strategies such as the selection of most potent CAR T cell precursors prior to manufacturing, arming the CAR-engineered cells with “disruptive effector molecules,” combinations of CAR T cells with other treatment modalities, and multi-antigen targeting are being investigated in various pre-clinical studies, hoping to melt more ice of limitations with an aim of achieving a cure of MM after CAR-T cell therapy.

## References

[1] Carpenter RO Evbuomwan MO Pittaluga S B-cell maturation antigen is a promising target for adoptive T-cell therapy of multiple myeloma. *Clin Cancer Res* 2013;19 (8):2048–2060. 23344265 10.1158/1078-0432.CCR-12-2422 PMC3630268

[2] Lin Y Raje NS Berdeja JG Idecabtagene vicleucel (ide-cel, bb2121), a BCMA-directed CAR T cell therapy, in patients with relapsed and refractory multiple myeloma: updated results from phase 1 CRB-401 study. *Blood* 2020;136 (Supplement 1):26–27.

[3] Madduri D Berdeja JG Usmani SZ CARTITUDE-1: phase 1b/2 study of ciltacabtagene autoleucel, a B-cell maturation antigen-directed chimeric antigen receptor T cell therapy, in relapsed/refractory multiple myeloma. *Blood* 2020;136 (Supplement 1):22–25.

[4] Gorovits B Koren E. Immunogenicity of chimeric antigen receptor T-cell therapeutics. *BioDrugs* 2019;33 (3):275–284. 31069709 10.1007/s40259-019-00354-5

[5] Wang D Wang J Hu G A Phase I study of a novel fully human BCMA-targeting CAR (CT103A) in patients with relapsed/refractory multiple myeloma. *Blood* 2021. 10.1182/blood.2020008936 33512480

[6] Gagelmann N Riecken K Wolschke C Development of CAR-T cell therapies for multiple myeloma. *Leukemia* 2020;34 (9):2317–2332. 32572190 10.1038/s41375-020-0930-x

[7] Xu J Chen LJ Yang SS Exploratory trial of a biepitopic CAR T-targeting B cell maturation antigen in relapsed/refractory multiple myeloma. *Proc Natl Acad Sci USA* 2019;116 (19):9543–9551. 30988175 10.1073/pnas.1819745116 PMC6510991

[8] Raje N Berdeja J Lin Y Anti-BCMA CAR T-cell therapy bb2121 in relapsed or refractory multiple myeloma. *N Engl J Med* 2019;380 (18):1726–1737. 31042825 10.1056/NEJMoa1817226 PMC8202968

[9] Singh N Orlando E Xu J Mechanisms of resistance to CAR T cell therapies. *Semin Cancer Biol* 2020;65:91–98. 31866478 10.1016/j.semcancer.2019.12.002 PMC7684646

[10] Fraietta JA Lacey SF Orlando EJ Determinants of response and resistance to CD19 chimeric antigen receptor (CAR) T cell therapy of chronic lymphocytic leukemia. *Nat Med* 2018;24 (5):563–571. 29713085 10.1038/s41591-018-0010-1 PMC6117613

[11] Karschnia P Jordan JT Forst DA Clinical presentation, management, and biomarkers of neurotoxicity after adoptive immunotherapy with CAR T cells. *Blood* 2019;133 (20):2212–2221. 30808634 10.1182/blood-2018-12-893396

[12] Park JH Riviere I Gonen M Long-term follow-up of CD19 CAR therapy in acute lymphoblastic leukemia. *N Engl J Med* 2018;378 (5):449–459. 29385376 10.1056/NEJMoa1709919 PMC6637939

[13] Locke FL Ghobadi A Jacobson CA Long-term safety and activity of axicabtagene ciloleucel in refractory large B-cell lymphoma (ZUMA-1): a single-arm, multicentre, phase 1-2 trial. *Lancet Oncol* 2019;20 (1):31–42. 30518502 10.1016/S1470-2045(18)30864-7 PMC6733402

[14] Cappell KM Sherry RM Yang JC Long-term follow-up of anti-CD19 chimeric antigen receptor T-cell therapy. *J Clin Oncol* 2020;38 (32):3805–3815. 33021872 10.1200/JCO.20.01467 PMC7655016

[15] Cohen AD Garfall AL Stadtmauer EA B cell maturation antigen-specific CAR T cells are clinically active in multiple myeloma. *J Clin Invest* 2019;129 (6):2210–2221. 30896447 10.1172/JCI126397 PMC6546468

